# Land-cover change and urban heat in a warming climate: a global satellite perspective

**DOI:** 10.1007/s00704-026-06604-1

**Published:** 2026-10-10

**Authors:** Saghi Sarafi, Christopher D. Lippitt, Gernot Paulus

**Affiliations:** 1 Department of Geography and Environmental Studies, University of New Mexico, Albuquerque, NM 87131, USA; 2 Department of Geoinformation and Environmental Technologies, Carinthia University of Applied Sciences, Villach 9524, Austria; 3 UNM Center for the Advancement of Spatial Informatics Research and Education (ASPIRE), University of New Mexico, Albuquerque, NM 87131, USA

## Abstract

The Urban Heat Island effect intensifies urban temperatures and exacerbates climate-related warming, yet how land cover (LC) buffers or amplifies this warming remains poorly quantified. This study analyzed satellite-derived land surface temperature (LST) across 100 cities from 2000 to 2020 using summer Landsat imagery at five-year intervals, evaluating LC–LST relationships through a three-level framework: net compositional change, pixel-level transition-specific pathways, and a Relative Temperature Index (RTI) revealing cross-city thermal signatures. LC change does not simply raise average temperatures – it preferentially amplifies the most dangerous heat. Extreme-heat metrics responded 1.5–1.7× more strongly than mean ΔLST to the same transitions. Water loss showed the strongest association with ΔLST increases (+ 3.7–4.9 K regardless of destination class), while impervious expansion was the most consistent predictor (+ 2.9–4.2 K across source classes) and the strongest significant predictor of net city-scale warming (R^2^ ≈ 0.23), alongside a significant cooling association with cropland gain. Mean LST across LC classes rose 1.3–2.6× faster than global mean land-only temperature over the study period, and extreme-heat metrics rose 2.9–4.5× faster, indicating urban surfaces amplify background climate warming beyond LC conversion alone. RTI analysis confirmed consistent relative thermal ordering across cities: impervious covers were consistently warmer than city means (RTI = 1.011), water cooler (RTI = 0.988), and vegetation near-neutral (RTI = 1.000). These results – consistent across three analytical levels, underscoring the value of this hierarchical framework – show that mitigation strategies should prioritize protecting water bodies and limiting impervious expansion, evaluated against extreme-heat metrics rather than mean temperature alone.

## 1 Introduction

Urbanization and rising global temperature mean and variance (Morice et al. 2021) are converging to create unprecedented environmental, social, and economic challenges, including increased heat-related mortality, disruption of urban water and energy systems, and escalating costs of climate-related disasters (Revi et al. 2014) for cities worldwide. Urban areas are now home to over half the global population, projected to reach 68% by 2050 (United Nations, Department of Economic and Social Affairs, Population Division 2018), and account for a disproportionate share of global energy use and greenhouse gas emissions, with urban energy use projected to increase more than threefold by 2050 (Creutzig et al. 2015). The combined effects of urbanization and climate change are particularly pronounced in the urban heat island (UHI) effect, where city temperatures routinely exceed those of surrounding rural areas, especially during extreme heat events (National Oceanic and Atmospheric Administration 2024). UHI amplifies health risks and disproportionately affects vulnerable populations (Heo and Bell 2023), increases energy demand for cooling (Wang et al. 2022), and accelerates infrastructure degradation.

Beyond direct temperature impacts, UHI exacerbates other urban challenges: it increases vapor pressure deficit and increases evapotranspiration (ET), further stressing urban vegetation and water resources (Zipper et al. 2017); it worsens air pollution and alters local weather patterns (Climate Adaptation Platform 2024); and it compounds climate risks for marginalized communities, who often reside in the hottest neighborhoods with the least access to cooling and green space (Hoffman et al. 2020; U.S. Environmental Protection Agency 2025). Because land surface temperature (LST) is closely linked to near-surface air temperature and heat exposure, especially during clear-sky conditions, spatial patterns of elevated LST can serve as a practical proxy for where urban residents are most likely to experience dangerous heat (Mildrexler et al. 2011).

The interplay between the land-cover (LC) change that typically accompanies rapid urbanization and rising global temperatures and temperature extremes (i.e., variability) (Zhang et al. 2021) makes understanding and mitigating UHI drivers a priority for sustainable urban development and public health. UHI is known to be driven by factors such as the replacement of natural vegetation with impervious surfaces (e.g., asphalt, concrete) (Zhang et al. 2022), reduced ET (Rivera et al. 2024), and anthropogenic heat emissions (Shahmohamadi et al. 2011). Urban geometry, including narrow streets and tall buildings, traps heat and reduces airflow, intensifying the UHI effect (Ren et al. 2023).

LC plays a critical role in modulating surface temperatures – vegetated areas often remain cooler, while built-up zones absorb and retain more heat, though intra-class variability can be large (Geletič et al. 2019). The cooling effect of water bodies and vegetation is principally driven by increased ET, which transfers heat away from the surface but can also intensify local water demand, particularly under conditions of overall warming and more frequent heatwaves. The spatial distribution and type of LC directly influence the intensity and spatial pattern of UHI (Chen et al. 2006). Understanding these dynamics is essential for urban planning and climate adaptation strategies, including where to prioritize green and blue infrastructure to reduce heat exposure for at-risk populations.

Remote sensing has been vital to UHI research due to its ability to provide consistent, large-scale, and repeatable observations of LST and LC change (Almeida et al. 2021; NASA 2022). Thermal infrared sensors (TIRS) have been central to monitoring UHI, measuring emitted thermal radiation to estimate LST, and serve as a key indicator of UHI intensity and spatial variability across urban landscapes (Almeida et al. 2021). The ability to integrate thermal data with LC classifications enables measurement of how urbanization is impacting local climate and the lived experience of residents (Zhou et al. 2014). Previous studies have examined relationships between LC and LST at single points in time or over limited temporal windows, often focusing on individual cities or short-term dynamics using remote sensing approaches (Peng et al. 2012; Zhou et al. 2014; Chakraborty et al. 2019; Islam et al. 2021). Several case studies have also explored the influence of LC change on LST at local or regional scales (e.g., Wuhan and Beirut), demonstrating that urbanization processes can significantly alter surface thermal patterns (Chen et al. 2022; Khyami 2022; Zhou et al. 2014). Rather than characterizing UHI intensity patterns (e.g., Zhou et al. 2014), this study seeks to isolate how specific LC transitions mediate long-term urban warming. It does so by leveraging advances in remote sensing and cloud computing to provide a comprehensive, multi-decadal analysis of LST-LC dynamics across 100 major global cities.

Using annual summer-season Landsat imagery from 2000 to 2020, we quantify changes in LST by LC type – impervious, vegetation, cropland, water, and bare – derived from the Global Land Analysis and Discovery (GLAD) LC dataset (Potapov et al. 2022), to evaluate the impact of LC change on LST at five-year intervals. City-level analyses quantify aggregate LC and LST change within city administrative boundaries and pixel-level analyses track individual LC transitions to isolate the thermal response of each conversion pathway; the Relative Temperature Index (RTI) then captures generalizable cross-city thermal signatures independent of background climate. By comparing LST and LC over time, this research aims to improve understanding of how LC is mediating increasing temperature and temperature variability in cities to provide actionable insights to urban planners and policymakers seeking to design climate-resilient cities.

## 2 Methods

This study analyzes LC–LST relationships at three complementary levels: (1) net compositional change, which captures aggregate city-level thermal effects; (2) transition-specific pathways, which identify the mechanistic pathways underlying those effects at the pixel-level; and (3) RTI, which provides generalizable cross-city patterns.

### 2.1 Study area

This study analyzes 100 major cities across the Northern (n=73) and Southern Hemispheres (n=27), selected to maximize geographic representation (Fig. 1). Initial sampling prioritized cities by population ranking to capture the diversity of global urban environments, while additional emphasis was placed on including cities from regions that are typically underrepresented in large-scale studies, particularly in South America, Africa, and Oceania, to promote spatial balance and reduce bias toward highly urbanized continents like Europe and Asia. The cities span a broad range of latitudes, biomes, and cultural contexts. The selected cities range from approximately 60°N (Oslo) to about 42°S (Hobart), with a mean latitude of about 20°N, indicating a northern hemisphere bias in the sample. An Absolute mean latitude of ~25° indicates a broad distribution from the equator. City populations range from roughly 200,000 (e.g. Geneva in 2020) to over 20 million (e.g. Cairo and Beijing in 2020, Mumbai in 2015), with a mean of around 5 million residents, reflecting diverse urban scales from mid-sized regional centers to metropolitan areas (Ritchie et al. 2025).

### 2.2 Data

All data used in this study were accessed and processed through a cloud-based geospatial computing platform, Google Earth Engine (GEE) (Gorelick et al. 2017), which enables efficient retrieval, integration, and analysis of satellite imagery and spatial datasets at global scale.

#### 2.2.1 Land cover

LC information was obtained from the Global Land Analysis and Discovery (GLAD) LC dataset (Potapov et al. 2022), which offers annual LC classifications at 30-meter resolution for multiple years, including 2000, 2005, 2010, 2015, and 2020. All data access, reclassification, and aggregation steps were performed using GEE, enabling integration with other spatial datasets. The original GLAD classification was reclassified into five thematic categories (Table 1): Impervious, Vegetation, Cropland, Water, and Bare. This scheme builds on established approaches (Zhang et al. 2024) but is modified to better capture urban thermal contrasts from a physical and urban-planning perspective. Specifically, impervious surfaces were separated from bare and vegetation classes to differentiate materials with distinct albedo and ET potential.

#### 2.2.2 Land surface temperature data

LST data were derived from the thermal infrared bands of Landsat 5 Thematic Mapper (TM), Landsat 7 Enhanced Thematic Mapper Plus (ETM+), and Landsat 8/9 Operational Land Imager (OLI) and TIRS systems, covering the period from 2000 to 2020. All satellite imagery retrieval and preprocessing, including data access, cloud masking, and resampling, were conducted in GEE.

Thermal bands, natively acquired at 120 m (Landsat 5 TM), 60 m (Landsat 7 ETM+), and 100 m (Landsat 8/9 TIRS), were standardized to 30 m spatial resolution using bilinear interpolation. LST was computed with a modified version of the NDVI-based emissivity correction and atmospheric adjustment method developed by Ermida et al. (2020). This implementation yields typical uncertainties of ± 1 K under clear-sky conditions. Sensor selection was dynamic: Landsat 5 was used through 2011, Landsat 7 for 2012, and Landsat 8/9 from 2013 onward, ensuring temporal continuity.

To represent UHI conditions, imagery was limited to the local summer season from June 1 to August 31 for Northern Hemisphere cities and November 1 to March 31 for Southern Hemisphere cities. The Southern Hemisphere window is wider than the Northern window to compensate for higher regional cloud cover during the Southern summer season, ensuring sufficient valid scenes for reliable LST retrieval; only cloud-free, quality-filtered scenes within each window contribute to the final per-city-year LST estimates. Clouds, shadows, and low-quality pixels were masked using the NASA-provided quality assurance (QA) bands distributed with each Landsat scene and ingested by GEE, following Ermida et al. (2020) protocols. Only scenes with valid LST retrievals were retained, city-year-LC combinations with no valid pixels were excluded (i.e., the GEE reducer returned null). ENSO years were classified using the NOAA Climate Prediction Center’s Oceanic Niño Index (ONI), with El Niño years defined as ONI≥+0.5 °C and La Niña years as ONI≤−0.5 °C, sustained for at least five consecutive overlapping three-month seasons, following standard NOAA CPC classification.

For each city polygon, delineated using administrative boundary polygons retrieved from OpenStreetMap via the OSMnx Python package (Boeing 2017; OpenStreetMap contributors 2024), summary statistics were calculated for each analysis year (2000, 2005, 2010, 2015, 2020) and per LC class: (1) mean LST (LST), (2) maximum LST (LST_Max_), and (3) mean of the upper-decile (LST_Max10_), computed as the mean of the top 10% of LST pixel values within each city-year-LC combination. These statistics are aggregated at the city-level; pixels are used only to define LC classes and LC transitions. The resulting dataset contains one row per city × year × LC class or class transition, with LST, LST_Max_, and LST_Max10_ for each. These metrics are summarized in Table 2.

As a global point of comparison for city-level warming trends, global mean land-only surface temperature (2000–2020) was obtained from Berkeley Earth’s land-only TAVG product (Rohde and Hausfather 2020), a station-based reconstruction that reports both temperature anomalies and absolute values relative to a 1951–1980 baseline (baseline absolute temperature: 8.59±0.04 °C). Annual absolute values were derived by adding each year’s annual anomaly to this baseline.

### 2.3 Hierarchical analytical framework for LC–LST analysis

This study analyzes LC–LST relationships at three complementary levels:

Level 1 – Net LC compositional change, which employed ordinary least squares (OLS) regression models to quantify how net percentage changes in each LC class between 2000 and 2020 explain city-level changes in LST, LST_Max_, and LST_Max10_. This level answers questions like, e.g., *Do cities that increase impervious cover experience more warming?*

Level 2 – Transition-specific pathways (mechanistic pathways at pixel-level), where pixel-level tracking of individual LC conversions (e.g., veg→imp) provides direct empirical measures of thermal change associated with each pathway. This level answers: *What specific LC conversions are associated with warming or cooling, and how large are these effects?* ΔLST was computed only within pixels that experienced the specific transition under consideration, isolating the thermal signal of each conversion pathway.

Level 3 – RTI (generalizable cross-city patterns), where RTI normalizes class-specific LST by mean LST at the city-level, revealing consistent thermal signatures that transcend climatic and geographic context. This level answers: *Are the observed LC-LST patterns persistent across cities, regardless of climate?* While RTI is not intended as a predictive planning tool, it provides a standardized way to compare the relative thermal behavior of LC types across cities, offering insight into which surfaces are consistently warmer or cooler than their urban context.

#### 2.3.1 Statistical analysis of net LC predictors of LST change – level 1

To quantify LC contributions to city-level warming in the general case, OLS multiple regression models were fitted at the city-level between city-level change in mean summer LST between 2000 and 2020 $\left(\Delta LST_{00\to 20}\right)$. Predictor variables consisted of net percentage changes in LC classes over the same period $\left(\Delta \%LC_{i,00\to 20}\right)$. All five LC predictors (i=1…5 index of the five LC classes) were entered simultaneously to estimate their independent contributions while controlling for compositional overlap among classes. The regression model is then:

(1) $$\Delta LST=\beta_{0}+\sum_{i=1}^{5}\beta_{i}.\Delta \%LC_{i,00\to 20}+\epsilon$$

Where $\beta_{0}$ is the intercept, ϵ is the residual error, and $\beta_{i}$ is the regression coefficient representing the contribution of LC class i to ΔLST (units: K per percentage-point LC change). The city-level thermal response variable ΔLST is defined as:

(2) $$\Delta LST_{00\to 20}=LST_{2020}-LST_{2000}$$

A single 2000–2020 endpoint comparison, rather than four successive 5-year differences, was used for this net-change analysis to isolate the long-term LC-associated signal from the interannual, ENSO-related variability visible in the 5-year time series (Fig. 3), which would otherwise dominate shorter-interval difference estimates. Parallel models were fitted for ΔLST_Max_ and ΔLST_Max10_ as response variables, defined analogously to Eq. 2, to capture how LC-associated thermal effects extend to peak and extreme heat conditions.

Models were implemented using the “statsmodels” OLS framework (Seabold and Perktold 2010). Predictors were not standardized to preserve physical interpretability of coefficients (β in K per % LC change). Model performance was evaluated using R^2^, adjusted R^2^, P-value, and coefficient-level t-tests (α=0.05). Variance inflation factors and condition numbers were examined to assess multicollinearity. Because the five LC classes represent percentages of a fixed total city area, their net changes are compositionally constrained: by construction, $\Delta \%LC_{\text{imp}}+\Delta \%LC_{\text{veg}}+\Delta \%LC_{\text{crop}}+\Delta \%LC_{\text{wat}}+\Delta \%LC_{\text{bare}}=0$ for every city, so an increase in one class’s cover is necessarily offset by a decrease elsewhere. This structural interdependence, rather than incidental correlation, is the primary source of collinearity among predictors in this model. Coefficients were therefore interpreted conservatively, as reflecting the relative contribution of each class conditional on the others, and in conjunction with the transition-specific analyses (Level 2), which examine each conversion pathway directly rather than relying on net compositional change alone. Bivariate correlation analyses preceding the regression models were additionally evaluated using Benjamini-Hochberg correction for multiple comparisons (Benjamini and Hochberg 1995), given the number of correlation tests conducted within each table.

Two boundary-sensitivity covariates were added to the Level 1 regression models to test whether administrative boundary characteristics confound the net-change results. The first, an area ratio, was calculated for each city as the ratio of its administrative boundary’s land area to the area of the corresponding population/built-up-defined “Urban Centre” extent from the Global Human Settlement Layer’s Degree of Urbanization settlement grid (GHS-SMOD; Schiavina et al. 2023), which delineates urban extent independent of administrative definitions. The second, a perimeter-area ratio (perimeter divided by the square root of area), quantifies boundary shape irregularity, with lower values indicating more compact, circular boundaries. Both covariates were computed in GEE and added to the existing five-predictor models described above. Figure 2 shows the resulting distribution of area ratios across the study sample: while the median city’s boundary closely approximates its GHS-SMOD-defined urban extent (median = 1.14), administrative boundaries vary substantially in how well they represent each city’s true urban extent, ranging from considerably undersized (ratio as low as 0.03) to considerably oversized (ratio as high as 31.3).

#### 2.3.2 LC transition analysis – level 2

To identify the mechanistic pathways underlying net changes, a pixel-level transition analysis was conducted. LC transitions between 2000 and 2020 were derived by overlaying LC maps for both years within each city boundary. For each pixel, its LC class in 2000 and 2020 was recorded. For each city and for each transition type i→j, the associated thermal response ΔLST was calculated as the mean difference between 2020 LST and 2000 LST across all pixels of that transition type:

(3) $$\Delta LST_{i\to j}=\text{mean}\left(LST_{2020,\text{destination}}-LST_{2000,\text{source}}\right)$$

A transition was considered to have sufficient cross-city representation if it occurred in at least 6 cities (i.e., more than the rare-transition threshold of ≤ 5 cities). Transitions with n≤5 cities are noted as rare and interpreted with caution.

This pixel-level approach yields a direct empirical measure of the thermal consequence of each LC conversion pathway (units: Kelvin) in the location of that transition. These pixel-level thermal responses are then aggregated to the city-level by summarizing, for each transition type, the mean and standard deviation of $\Delta \text{LST},\Delta LST_{\text{Max}}$, and $\Delta LST_{\text{Max}10}$ across all cities where that transition occurred. This aggregation enables consistent cross-city comparison while avoiding inflation of statistical significance due to large pixel counts and spatial autocorrelation.

Direct thermal responses (Eq. 3) constitute the primary evidence base for Level 2; transition area is reported as a percentage of total city area for descriptive context only. A pixel-level statistical analysis of transition classes was not conducted because the study is designed around city-level inference, and pixel-level observations would be spatially autocorrelated.

#### 2.3.3 Relative temperature index – level 3

To enable standardized cross-city comparisons of LC thermal impacts, RTI was calculated for each city, year, and LC class using the aggregated city-level LST dataset. For every city-year, the mean LST for each LC type was first computed from the Landsat-derived data, then normalized by the city-year mean LST across all five LC classes. The RTI for LC class i in city c and year t is defined as:

(4) $$RTI_{c,t,i}=LST_{c,t,i}/LST_{c,t}$$

Values greater than 1.0 indicate that a LC type is warmer than the city-year average (contributing to city-level warming), whereas values below 1.0 indicate cooling relative to the city average. RTI was computed for all available city-year-LC combinations in the dataset (n=2244 across 100 cities). To minimize mixed-pixel effects, the lower and upper 10% of values (10th and 90th percentiles) were excluded when reporting min/max RTI values. Mean, standard deviation, and mode were calculated using all available data.

## 3 Results

This section presents findings across the three analytical levels outlined in the Methods: (1) net LC change and its relationship to city-level LST trends; (2) pixel-level transition-specific thermal responses; and (3) city-level RTI.

### 3.1 Temporal dynamics of LST and LC (2000–2020)

LST trends by LC (Fig. 3) confirms that, on average, the impervious class has the highest LSTs across all years, followed by cropland and vegetation; the water class consistently remain coolest, reinforcing its role as an urban thermal buffer. For extreme heat (i.e., $\Delta LST_{\text{Max}}$, and $\Delta LST_{\text{Max}10}$), impervious and vegetation classes show amplified differences compared to mean conditions. All classes experienced rapid warming through 2000–2015, after which a peak in LST is synchronously observed near 2015, coinciding with the major El Niño event of 2015–2016, followed by stabilization or slight cooling. Over the study period, mean LST across urban LC classes increased by roughly 1.2–2.3 K, compared to an increase of 0.90 K in global mean land-only temperature (Berkeley Earth land-only TAVG; Rohde and Hausfather 2020) over the same interval – 1.3–2.6× the global rate. Extreme-heat metrics rose by 2.7–4.1 K, or 2.9–4.5× the global rate – cities are warming several times faster than land generally, and the gap widens further for extreme heat. Vertical ENSO reference lines are shown for context; because LST was sampled at five-year intervals (2000, 2005, 2010, 2015, 2020), the dataset is not resolved at the temporal scale needed to associate individual ENSO events with specific interannual fluctuations, and no such association is inferred here.

Over the two-decade study period, LC composition shifted systematically across the 100-city sample (Fig. 4). Impervious cover increased steadily from a mean of 55.4% in 2000 to 63.0% in 2020, while vegetation declined from 26.2% to 21.7% over the same interval. Cropland also decreased, from 7.5% to 5.9%, whereas water and bare cover remained relatively stable, each accounting for less than 8% of urban area throughout the period. Individual city trajectories reveal considerable heterogeneity – some experienced rapid impervious expansion exceeding 10% points over two decades, while others showed little change or even slight vegetation recovery – yet the directional trends of impervious gain and vegetation loss were consistent across diverse global contexts.

LC fractions vary widely across the sample (impervious: 2–100%; vegetation: 0–92%), reflecting genuine differences in administrative boundary extent and urban morphology rather than data artifacts. Cities with compact drawn boundaries – such as Santiago, Athens, and Paris – show near-complete impervious cover (>97%), while cities with larger boundaries that include suburban land – such as Kinshasa, Brasília, and Melbourne – show high vegetation fractions. In a few cases, such as Baku City, the boundary also includes a large water area, so the water fraction reflects boundary geometry rather than urban water infrastructure. These are known edge effects of using standardized administrative boundaries in global multi-city studies (Boeing 2017).

Figure 5 displays the relationships between the percent cover of five LC classes and mean summer LST in 2020. Higher impervious fractions show no significant association with LST (r=0.0589,p=0.5624,n=99). Greater vegetation fractions show modest cooling (r=-0.2220,p=0.0272,n=99) and bare cover shows modest warming (r=0.2397,p=0.0237,n=89). Correlations for cropland (r=0.0569,p=0.5837,n=95) and water (r=-0.1378,p=0.1784,n=97) are small and non-significant. As shown in Table 3, vegetation’s cooling association does not extend to $LST_{\text{Max}}$, and $LST_{\text{Max}10}$ (both p>0.07), and bare cover shows nominal warming across all three metrics (LST: $r=0.2397,p=0.0237;LST_{\text{Max}}:r=0.2226,p=0.0360;LST_{\text{Max}10}:r=0.2236,p=0.0352$); however, none of these associations survive Benjamini-Hochberg correction for multiple comparisons (all corrected p≥0.135). Across all classes, |r|≤0.24, reflecting the dominant influence of regional climate, elevation, and other non-LC factors on city-scale LST. Because comparing cities against each other conflates LC with these confounding regional differences, static composition alone is not a reliable predictor of a city’s absolute temperature. These weak, non-robust static relationships instead motivate the change- and transition-based analyses that follow, which compare each city against its own baseline and thereby control for background climate, latitude, and elevation directly.

### 3.2 Influence of LC change (2000–2020) on ΔLST –level 1: net compositional change

Figure 6 illustrates how urban LC changes from 2000 to 2020 $\left(\Delta LC_{i,00\to 20}\right)$ relate to corresponding changes in mean summer $\Delta \text{LST}\left(\Delta LST_{00\to 20}\right)$ at the city-level. Increases in impervious cover was associated with significant LST increases (r=0.3203,p=0.0054). Decreases in cropland showed a strong and highly significant correlation with warming (r=-0.3826,p=0.0008), indicating that cities losing cropland experienced greater warming. Relationships for water, vegetation, and bare cover changes showed weaker and statistically non-significant associations with $\Delta LST_{00\to 20}$. For extreme heat metrics, the impervious warming signal remains significant ($\Delta LST_{\text{Max}}:r=0.2980,p=0.0099;\Delta LST_{\text{Max}10}:r=0.2996,p=0.0095$). The cropland signal also persists and remains significant under extreme conditions ($\Delta LST_{\text{Max}}:r=-0.2655,p=0.0222;\left.\Delta LST_{\text{Max}10}:r=-0.2683,p=0.0208\right)$. Water cover change shows a modest but significant cooling association with extreme heat $\left(\Delta LST_{\text{Max}}:r=-0.2448,p=0.0355\right.;\left.\Delta LST_{\text{Max}10}:r=-0.2510,p=0.0310\right)$. Table 4 reports both uncorrected and Benjamini-Hochberg-corrected p-values; the impervious associations survive correction across all three metrics, while cropland survives only for mean LST. These bivariate correlations motivate, but are not the primary evidence for, the multivariate regression results presented below, which more directly isolate the independent contribution of each LC class.

Multiple regression analysis, including two boundary-sensitivity covariates (area ratio and perimeter-area ratio) alongside the five LC predictors, confirmed that net LC change explains a modest but significant share of cross-city warming variability R^2^ = 0.2270, adjusted R^2^ = 0.1534, p=0.0203,n=70). Impervious expansion was the strongest positive predictor of warming (β=0.1884,p=0.0096), and cropland change exhibited a significant negative association with ΔLST(β=-0.2072,p=0.0398), indicating that cities that gained cropland tended to remain cooler, while those that lost cropland warmed more. This cropland effect was significant only for mean LST, not for extreme heat (β=-0.1022,p=0.4368 for $\Delta LST_{\text{Max}};\beta =-0.1022,p=0.4342$ for $\Delta LST_{\text{Max}10}$). Net changes in vegetation, water, and bare surfaces were not significant predictors of $\Delta LST_{00\to 20}$ (all p>0.27), likely reflecting multicollinearity among compositional LC fractions. Parallel models for $\Delta LST_{\text{Max}}$, and $\Delta LST_{\text{Max}10}$ showed broadly consistent patterns, with impervious expansion remaining the strongest positive predictor and its coefficient growing larger under extreme heat $\left(\Delta LST_{\text{Max}}:\beta =0.2989,p=0.0021\right.;\Delta LST_{\text{Max}10}:\beta =0.3005,p=0.0019)$, indicating that impervious expansion disproportionately intensifies peak heat. Neither boundary covariate was a significant predictor (area_ratio: β=-0.0134,p=0.8656; perimeter_area_ratio: β=-0.1592,p=0.1945), indicating that the core findings are not confounded by administrative boundary representativeness or shape. One city (Baku) was excluded from this analysis after a data-quality check revealed that its administrative boundary includes a substantial area of the Caspian Sea, reducing the sample to 73 cities. Of those, three cities (Brasília, Hobart, and National Capital District) also lacked a usable area-ratio value and were excluded, giving a final sample of 70 cities for this analysis (see Limitations for further discussion). Full results are reported in Table 5.

### 3.3 Transition-specific ΔLST responses - Level 2: mechanistic pathways

Table 6 reports pixel-level thermal responses by transition type. Sample sizes vary substantially across transitions (Table 6, n_cities column), ranging from as few as 2 cities (imp→crop) to more than 90 (veg→imp); transitions observed in ≤5 cities are flagged in Table 6 and should be interpreted with particular caution throughout the results and discussion below. These results reinforce the city-level findings: conversions to impervious surfaces consistently produce warming and that water loss shows the strongest association with surface temperature change across all destination classes (Table 6). Across the 92 cities that had both valid LST retrievals for 2000 and 2020 and at least one LC transition pixel, the average city-level ΔLST was +2.140 K; $LST_{\text{Max}}$ increased by +3.899 K and $LST_{\text{Max}10}$ by +3.856 K. This gap – extreme heat metrics warming nearly twice as much as the mean – confirms that LC change disproportionately intensifies peak heat exposure, one of the central findings of this study.

All conversions to impervious surfaces are associated with substantially higher LST across all three LST metrics (mean LST: veg→imp+2.8571 K, crop→imp+3.2462 K, wat→imp+4.1619 K, bare→imp+3.0448 K). $LST_{\text{Max}}$ responses are approximately 1.6× larger for water-loss transitions (e.g., wat→imp: +6.0686 K vs. +4.1619 K mean) and range from 1.5–1.7× for impervious expansion: veg→imp+4.8059 K (ratio 1.68), crop→imp+5.1415 K (1.58), wat→imp+6.0686 K (1.46), bare→imp+4.4748 K (1.47), with $LST_{\text{Max}10}$ nearly identical in all cases.

Among non-impervious transitions, water loss is consistently associated with the strongest warming regardless of destination class, amplified for heat extremes. For mean LST, wat → bare warmed most (+4.9336 K), followed by wat→imp (+4.1619 K), wat→crop (+3.8838 K), and wat→veg (+3.7427 K). The transition analysis also reveals that cropland loss to vegetation produces warming (+2.4205 K), indicating that the thermal consequence of losing cropland is not solely a function of urbanization. This finding is consistent with recent global analyses showing that croplands can be cooler than surrounding natural ecosystems in many contexts, meaning their replacement – even by vegetation – can result in warming (Chen et al. 2024). This suggests that the biophysical properties of cropland – whether related to aerodynamic resistance, leaf area index, albedo, or ET – confer a cooling effect that is diminished when cropland is replaced by other LC types. By contrast, cropland loss to water produces near-neutral cooling (−0.41 K), underscoring water bodies’ unique thermal moderating role. $LST_{\text{Max}}$ responses for these transitions are larger: wat→bare (+6.6669 K), wat→crop (+6.3919 K), wat→imp (+6.0686 K), and wat→veg (+5.8891 K), with $LST_{\text{Max}10}$ values nearly identical. One minor anomaly was observed for the wat→crop transition, where $LST_{\text{Max}10}$ (+6.4235 K) slightly exceeded $LST_{\text{Max}}$ (+6.3919 K). This likely reflects ordinary statistical noise from averaging single-city $LST_{\text{Max}}$ and $LST_{\text{Max}10}$ values across cities with varying pixel counts for this transition; the difference (0.03 K) is well within the typical±1 K uncertainty of the Landsat LST retrieval (Ermida et al. 2020) and does not affect any substantive conclusion. The amplification ratio for water-loss transitions $\left(\Delta LST_{\text{Max}}/\right.\text{mean}\left.\approx 1.6\right)$ is among the largest observed for transitions with substantively large mean effects (excluding transitions with near-zero mean ΔLST, where ratios are statistically unstable).

Transitions away from impervious cover are very rare in this dataset (fewer than 5 cities exhibited imp→veg or imp→crop transitions). These values should not be interpreted as reliable estimates of de-sealing cooling effects due to small-sample noise.

### 3.4 Relative temperature index – level 3: generalizable cross-city patterns

Having established the mechanistic pathways driving LC-related warming at the pixel level, Level 3 asks whether these thermal signatures generalize across climatically diverse cities. Across all city-years (n=2,244), RTI values reveal consistent patterns by LC class (Table 7; Fig. 7).

Impervious cover averaged RTI=1.0106 (SD=0.0069), systematically warmer than city averages and contributing to urban warming. Water showed the strongest cooling effect (RTI=0.9880, SD=0.0088), while vegetation was nearly neutral (RTI=1.0000, SD=0.0065). Cropland (RTI=1.0054, SD=0.0080) and bare land (RTI=0.9956, SD=0.0130) exhibited intermediate thermal signatures, with bare class showing greater variability across cities, likely reflecting differences in soil properties, moisture availability, and albedo. Because RTI normalizes each class’s LST to the city-year mean, its value could in principle depend on a city’s LC composition. To test this directly, RTI for each class was regressed against that class’s own percent cover across all city-years (Fig. 8). Impervious RTI increased significantly with %impervious cover (r=0.258,p<0.0001,n=463), and water RTI decreased significantly with %water cover (r=-0.274,p<0.0001,n=454) – in both cases, a larger share of the class within a city was associated with a stronger, not weaker, deviation from the city mean. This indicates that some other, real-world covariance between LC composition and local thermal conditions outweighs the mathematical dilution effect inherent to RTI’s normalization. One plausible contributor is spatial configuration: cities with a greater share of impervious cover typically have proportionally less vegetation and water, reducing the local cooling influence of these classes and likely resulting in larger, more contiguous impervious surfaces with less interspersed cooling than in less impervious cities. Vegetation showed a weaker but significant negative relationship (r=-0.122,p=0.009), while cropland and bare land showed no significant compositional dependence (both p>0.59). This pattern runs counter to a simple compositional-dilution expectation, meaning RTI magnitude should not be compared directly across cities with very different LC compositions without accounting for this relationship.

These tight standard deviations (0.0065–0.0130) across climatically diverse cities demonstrate robust, generalizable LC thermal performance: impervious surfaces are consistently warmer than city means, water cooler, and vegetation thermally neutral.

## 4 Discussion

This study examined how LC mediates urban LST dynamics across 100 global cities (2000–2020) using the three-level framework introduced above. The discussion below moves from aggregate outcome (Level 1) to mechanism (Level 2) to generalizable cross-city pattern (Level 3).

At the aggregate level (Level 1), net LC change explains a modest but significant share of cross-city warming variability (R^2^ = 0.2270, p=0.0203). This result was robust to the inclusion of boundary covariates (area ratio and compactness), which were not significant predictors and did not materially alter the impervious or cropland coefficients. Impervious expansion emerges as the dominant predictor (β=+0.1884,p=0.0096), consistent with a broad literature documenting the thermal consequences of surface sealing (Chen et al. 2006; Peng et al. 2012; Zhou et al. 2014). Impervious cover replaces materials with higher albedo and greater ET capacity with low-albedo, low-permeability surfaces that absorb and store heat, reducing latent heat flux and increasing sensible heat flux (Zhang et al. 2022). Critically, this impervious signal intensifies for extreme heat metrics (β=+0.2989 for ${LST}_{Max}$ and + 0.3005 for $LST_{\text{Max}10}$, both p<0.003), indicating that impervious expansion does not merely raise average temperatures but disproportionately amplifies peak heat exposure – the most climatically and epidemiologically consequential outcome.

A notable nuance within the Level 1 results is the significant negative coefficient for cropland change (β=-0.2072,p=0.0398): cities that gained cropland experienced lower mean LST, while those that lost cropland warmed more. This result likely reflects two complementary mechanisms. First, cropland loss is not uniformly associated with urbanization: while some cities lose cropland to impervious surfaces (producing warming), others lose it to vegetation or water (producing cooling), and these opposing pathways partially offset one another at the city scale. Second, a biophysical mechanism may be at work: recent satellite-based analyses have shown that croplands exhibit distinct thermal behavior compared to natural ecosystems, with 60% of global croplands showing a warming effect and 40% showing a cooling effect relative to surrounding vegetation (Chen et al. 2024). Aerodynamic resistance – which modulates latent heat flux – has been identified as the dominant biophysical factor controlling cropland LST, with the magnitude of the effect depending on differences in leaf area index and background climate (Chen et al. 2024). Where actively managed or irrigated, cropland may additionally sustain higher ET rates than some urban vegetation types, further suppressing surface temperatures through elevated latent heat flux (Kalnay and Cai 2003; Lobell et al. 2008). However, because the GLAD LC data do not distinguish irrigated from rainfed cropland, this irrigation-mediated cooling effect is inferred rather than directly measured. Alternative biophysical mechanisms – including higher albedo compared to darker urban surfaces and different aerodynamic roughness lengths – may also contribute to cropland’s distinct thermal signature (Zhao et al. 2014; Chen et al. 2024). When cropland is lost – whether to impervious cover or to vegetation with lower water availability – this ET-driven cooling diminishes, contributing to warming. It should be noted that the cropland effect is significant only for mean LST and does not extend to extreme heat metrics (β=-0.1233,p=0.347 for $LST_{\text{Max}};\beta =-0.1229,p=0.346$ for $LST_{\text{Max}10}$), suggesting that the mechanisms underlying cropland’s moderating influence on average temperatures may be less effective during heat extremes. Under high heat and water stress, many plants close their stomata to conserve water, which sharply reduces ET – consistent with known limitations of ET-based cooling under high vapor pressure deficit conditions (Seneviratne et al. 2010; Zipper et al. 2017). Net changes in vegetation, water, and bare surfaces are non-significant predictors (all p>0.17). This reflects the compositional structure of the predictors – because net changes in the five LC classes sum to zero by construction, any one class’s coefficient captures its contribution relative to the others, not in isolation – compounded by high cross-city heterogeneity in how these classes change. This structural interdependence is why the transition-specific analyses (Level 2), which isolate each conversion pathway directly, provide the more direct mechanistic evidence for this study’s conclusions.

The Level 2 transition analysis provides the mechanistic underpinning for these aggregate patterns. All conversions to impervious cover were associated with substantial warming regardless of source class (e.g., veg→imp: +2.86 K; crop→imp: +3.25 K; wat→imp: +4.16 K), confirming the dominant warming association of surface sealing. Water loss showed the strongest warming association of any transition type observed – larger than any impervious-expansion pathway – identifying it as the strongest thermal signal in this study. Among transitions with sufficient cross-city representation, crop→wat (−0.41 K mean ΔLST) was the only near-cooling signal observed – a conversion toward water, consistent with water bodies acting as thermal moderators rather than sources of warming. Transitions away from impervious surfaces are rare in the dataset (n≤5 cities) and show inconsistent signals. imp→crop produced a small negative mean ΔLST (−0.08 K), consistent with de-sealing reducing surface temperature. imp→veg shows a divergent pattern: mean LST warmed (+1.23 K) while extreme-heat metrics cooled ($LST_{\text{Max}}$ and $LST_{\text{Max}10}=-0.31\;\text{K}$), the opposite of the amplification pattern seen elsewhere in this study. imp→wat also warmed on average (+1.15 K) rather than cooling. Given how rare and spatially limited these transitions are within already built-up urban cores (e.g., small vegetation patches, new retention basins), all three are likely affected by edge and mixed-pixel effects and small-sample noise and should be treated as the least reliable estimates in Table 6. $LST_{\text{Max}}$ and $LST_{\text{Max}10}$ responses were systematically larger than mean LST responses, with water-loss transitions showing the strongest amplification $\left(LST_{\text{Max}}/\right.\text{mean}\left.\approx 1.6\right)$ and impervious expansion producing ratios of 1.5–1.7 for the same transitions (e.g., wat→imp: +6.07 K for $LST_{\text{Max}}$ vs. +4.16 K mean; veg→imp: +4.81 K for $LST_{\text{Max}}$ vs. +2.86 K mean), demonstrating that LC transitions amplify extreme heat disproportionately relative to mean warming.

The temporal LST trajectory (2000–2020) shows that all LC classes warmed in tandem over the study period. Because LST was sampled only at five-year intervals, this trajectory cannot resolve interannual variability, including short-term ENSO-driven fluctuations; the observed warming pattern instead reflects the longer-term trend across the study period. Over this period, impervious cover expanded steadily from a mean of 55.4% to 63.0% of city area, while vegetation declined from 26.2% to 21.7%. Cities that urbanized more rapidly and lost more pervious cover warmed systematically more over the two decades. Global mean land temperature increased by 0.90 K between 2000 and 2020 (Berkeley Earth land-only TAVG; Rohde and Hausfather 2020), while mean LST across LC classes increased by 1.2–2.3 K over the same period (1.3–2.6× the global rate), and extreme-heat metrics rose by 2.7–4.1 K (2.9–4.5× the global rate). This gap – evident even within LC classes that remained compositionally stable over the study period – indicates that urban warming cannot be explained by LC conversion alone. Instead, the intrinsic thermal properties of existing urban surfaces appear to amplify background climate warming, so both LC composition and LC transitions contribute to rising urban heat.

The RTI analysis (Level 3) confirms that these thermal roles generalize robustly across diverse cities and years. Impervious surfaces are consistently warmer than city means RTI = 1.0106, SD = 0.0069), water shows the strongest cooling effect (RTI = 0.9880, SD = 0.0088), and vegetation is nearly thermally neutral (RTI=1.0000, SD=0.0065). To assess whether these class-level differences are statistically distinguishable, paired t-tests were conducted between RTI distributions across city-years; all pairwise comparisons between LC classes were significant (p<0.001; 10 pairwise tests), surviving both Benjamini-Hochberg correction and the stricter Bonferroni-corrected threshold (α=0.005), confirming that the thermal separation between impervious, water, vegetation, cropland, and bare class is not attributable to sampling variance. Given the large sample size (n=2,244), however, statistical significance alone is a weak indicator of practical importance here; the more informative evidence is the magnitude of the RTI values themselves and their tight standard deviations (0.0065–0.0130), reported above. The tightness of these standard deviations across 100 cities spanning tropical, arid, temperate, and continental climates demonstrates that the relative thermal roles of each LC class are highly consistent across diverse climate zones, consistent with prior global analyses (Peng et al. 2012; Zhou et al. 2014). As detailed above (Fig. 8), this relative ordering is robust even though RTI magnitude itself is not fully independent of a city’s LC composition. The near-neutrality of vegetation (RTI≈1.0) does not imply that vegetation provides no absolute cooling benefit, but rather that its thermal signature at the city scale is comparable to the overall city mean.

The implication is that preserving or expanding water and vegetation represents a reliable, climate-agnostic thermal buffering strategy, while impervious expansion is consistently associated with cities above their own thermal baselines over time. Beyond these substantive findings, the consistency of results across three independent analytical levels – net compositional change, transition-specific pathways, and climate-normalized RTI – demonstrates the value of this hierarchical framework as a transferable approach for disentangling LC effects on urban climate in future cross-city research.

### 4.1 Practical implications for urban planners

The quantitative results from this study provide the following evidence-supported recommendations (Table 8) for urban planners:

These strategies are particularly critical in rapidly urbanizing regions, where development can exacerbate heat exposure. Vegetation- and water-based solutions offer scalable, cost-effective approaches to integrate climate adaptation into urban development. Preserving existing water bodies and green cover, and preventing their conversion to impervious surfaces, is the most thermally consequential intervention available to urban planners. Because elevated LST is closely associated with the near-surface heat conditions that drive human heat exposure (Mildrexler et al. 2011), these interventions are particularly relevant to reducing the peak heat exposure responsible for the greatest disproportionate burden of heat-related mortality and morbidity (Guo et al. 2017; Lüthi et al. 2023).

### 4.2 Limitations and future directions

The GLAD LC scheme aggregates diverse impervious materials (e.g., asphalt vs. reflective concrete), masking albedo effects that could refine thermal predictions. This is a well-recognized limitation of broad LC classifications for urban thermal analysis: spectral mixture and sub-pixel heterogeneity of impervious materials mean that a single impervious class conflates materials with substantially different albedo and heat-storage properties (Wu and Murray 2003; Small 2003; Weng 2012). Future work should incorporate albedo-based subclasses to better capture variability in surface energy balance. The 30 m resolution of Landsat limits detection of small-scale urban features such as narrow streets or individual trees. Additionally, LST serves as a surface proxy that may diverge from near-surface air temperature under certain atmospheric conditions, depending on atmospheric stability, wind speed, and surface roughness (Mildrexler et al. 2011; Venter et al. 2021). While net LC changes and transitions explain a modest share of city-scale variance in ΔLST (R^2^ ≈0.23), omitted variables such as urban morphology, building height, city compactness, street geometry, and anthropogenic heat from energy use likely play important roles and warrant inclusion in future multi-variable models. Anthropogenic heat from energy use – including air conditioning, transportation, and industrial activity – may be a particularly important omitted driver, given that this source of heat scales directly with urbanization and population density and is not captured by LC classification alone; its exclusion here likely contributes to the residual variance in ΔLST not explained by LC change. Relatedly, Fig. 5; Table 3 compare LC composition across cities using existing administrative boundaries, which vary in how well they represent each city’s full urban extent; boundary representativeness and shape were incorporated directly into the Level 1 regression as covariates and were not significant predictors (Table 5). A fully consistent urban-footprint redefinition of city boundaries extracted using physical properties (e.g., night-time lights, population density) rather than political boundaries represents an important direction for future work.

As noted in Results, one city (Baku) was excluded from the Level 1 regression due to a boundary data-quality issue, reducing the sample from 74 to 73 cities. Of those remaining 73 cities, three (Brasília, Hobart, and National Capital District) also lacked a usable area-ratio value and were excluded, giving a final regression sample of 70 cities. Excluding Baku specifically changed no headline result (Level 1 R^2^ = 0.186 → 0.196 in the original 5-predictor model; all RTI values changed by <0.0003), and these exclusions reflect a limitation of the GHS-SMOD comparison dataset for smaller or lower-density cities, rather than any issue with these cities’ own boundary data. The Level 1 net-change analysis also compares single endpoint years (2000, 2020) that differ in ENSO phase (La Niña and El Niño, respectively); while this may modestly influence the absolute magnitude of reported ΔLST values, the urban-to-global warming ratios reported here are less sensitive to this effect, since both series share the same endpoint years.

Expanding the analytical framework to examine how stable LC classes (those unchanged over the study period) mediate the relationship between LC change and LST would help isolate the modifying role of background urban structure. A specific limitation of the cropland interpretation is that the GLAD LC data do not distinguish irrigated from rainfed agriculture. As a result, our inference that irrigation-mediated ET contributes to cropland’s cooling effect is circumstantial rather than directly measured. Future work should explicitly test whether the thermal response to cropland loss varies by aridity index and irrigation intensity, as the cooling signal may be strongest in semi-arid cities where irrigated agriculture represents a disproportionate source of latent heat flux. The Global Aridity Index and Potential Evapotranspiration Database (CGIAR-CSI; Zomer et al. 2008) or WorldClim bioclimatic variables (Fick and Hijmans 2017) could be used to stratify cities by water availability and test this hypothesis directly. More broadly, recent advances in understanding the biophysical effects of croplands on LST (Chen et al. 2024) provide a foundation for future studies to disentangle the contributions of aerodynamic resistance, leaf area index, and background climate to cropland-mediated urban warming. Incorporating contextual variables such as country income level, aridity index, latitude, and elevation could illuminate why the strength of LC–LST relationships vary across cities and guide more targeted adaptation strategies—questions that the framework developed here is well-suited to address.

## 5 Conclusion

LC change disproportionately amplifies extreme heat, and water loss and impervious expansion emerge as the strongest transition pathways driving that amplification. By integrating analyses of net compositional change, transition-specific pathways, and climate-normalized thermal signatures across 100 global cities, we show that water loss is the strongest thermal association with warming, while impervious expansion remains the most consistent predictor of city-scale temperature increases. These findings suggest that effective urban heat mitigation requires protecting existing blue infrastructure, limiting surface sealing, and evaluating interventions based on their ability to reduce extreme heat rather than mean temperature alone.

At Level 1, net LC change explained a modest but meaningful share of cross-city ΔLST variability (R^2^ = 0.2270, p=0.0203), with impervious expansion the dominant predictor and cropland loss a secondary one, reflecting the heterogeneity of cropland conversion pathways and the ET-driven cooling that irrigated agriculture provides relative to the surfaces that replace it. At Level 2, all conversions to impervious surfaces produced consistent warming, while water loss showed the strongest signals observed regardless of destination class and extreme heat responses to these transitions were 1.5–1.7× larger than mean warming. At Level 3, the RTI confirmed that these thermal roles are highly consistent across cities spanning tropical, arid, temperate, and continental climates: impervious surfaces reliably warmer than city means, water reliably cooler, and vegetation near-neutral.

Taken together, these three levels tell a consistent story: impervious expansion is the most consistent lever available to planners, water loss is the most powerful one, and both show stronger associations with the hottest, most dangerous conditions than with the average. Priorities for mitigation should include restricting impervious expansion – particularly from cropland and water – preserving existing blue-green infrastructure and evaluating interventions against peak and upper-decile heat rather than mean warming alone.

## Figures and Tables

**Fig. 1:**
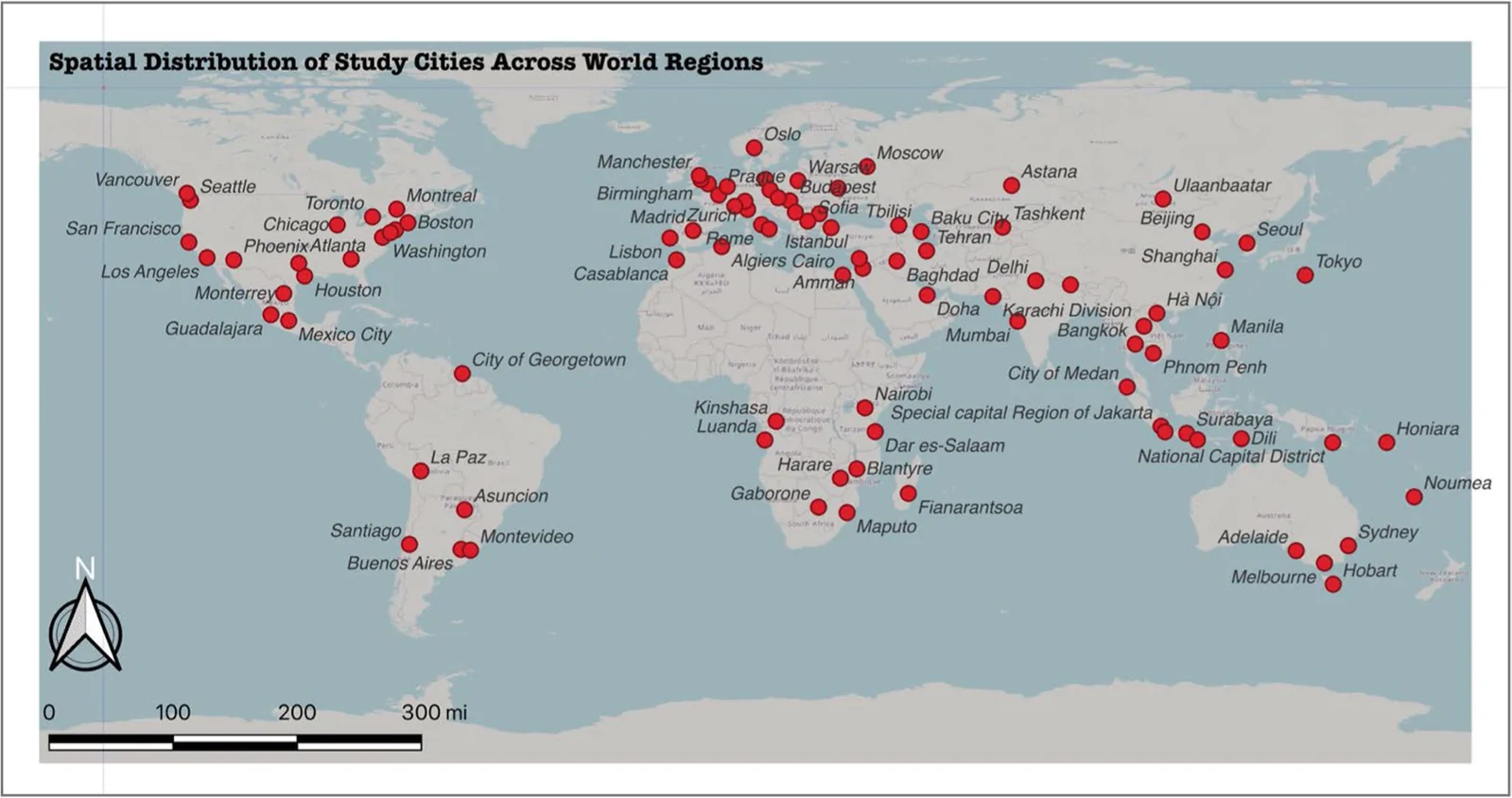
Global distribution of study cities. Red points represent the locations of cities included in the analysis, illustrating the geographic coverage of the study sample across world regions

**Fig. 2:**
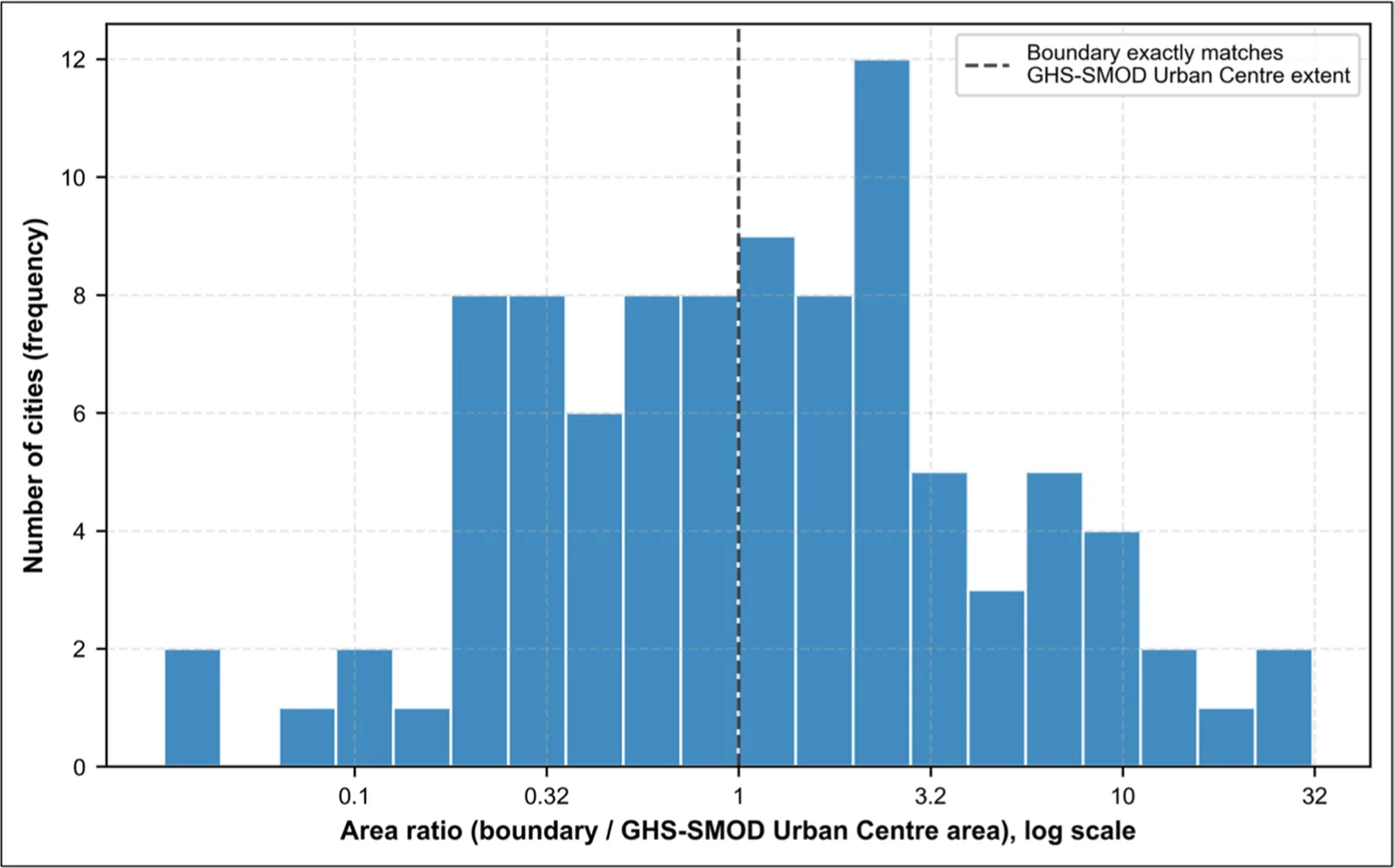
Distribution of the area-ratio boundary covariate (administrative boundary land area divided by GHS-SMOD Urban Centre extent) across the 95 study cities with a usable value. Values near 1.0 indicate a close match between the administrative boundary and the population/built-up-defined urban extent; values below 1.0 indicate the boundary is smaller than the true urban extent, values above 1.0 indicate it is larger. X-axis shown on a log scale given the long-tailed distribution (range: 0.03–31.3)

**Fig. 3:**
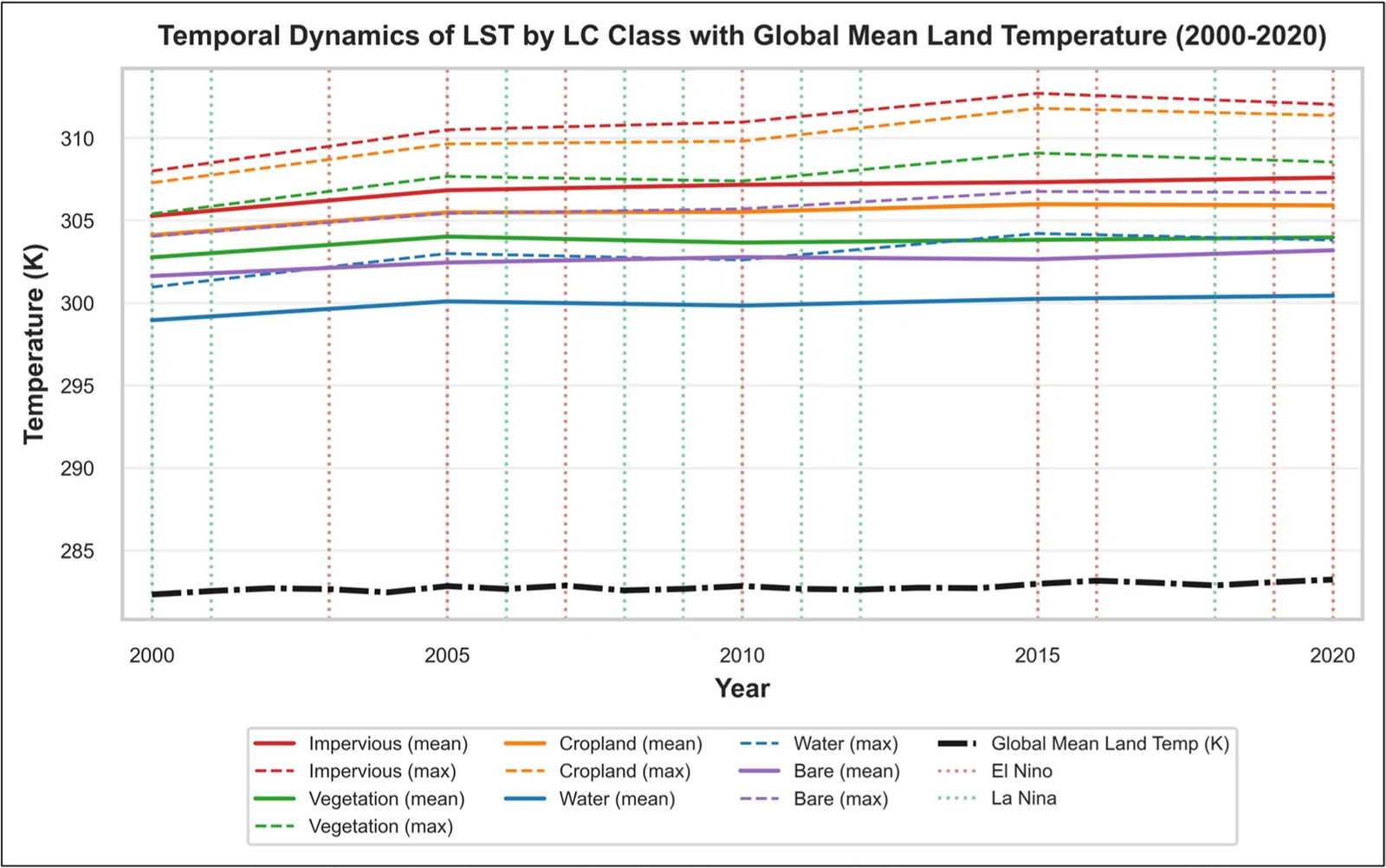
Trends of mean (solid) and maximum (dashed) LST for major LC classes (2000–2020, color-coded by class). Global mean land-only temperature (Berkeley Earth; Rohde and Hausfather 2020) is shown as a thick black dash-dot line. El Niño (red dotted lines) and La Niña (green dotted lines) years from NOAA CPC are indicated as vertical lines. All temperature values are in Kelvin

**Fig. 4:**
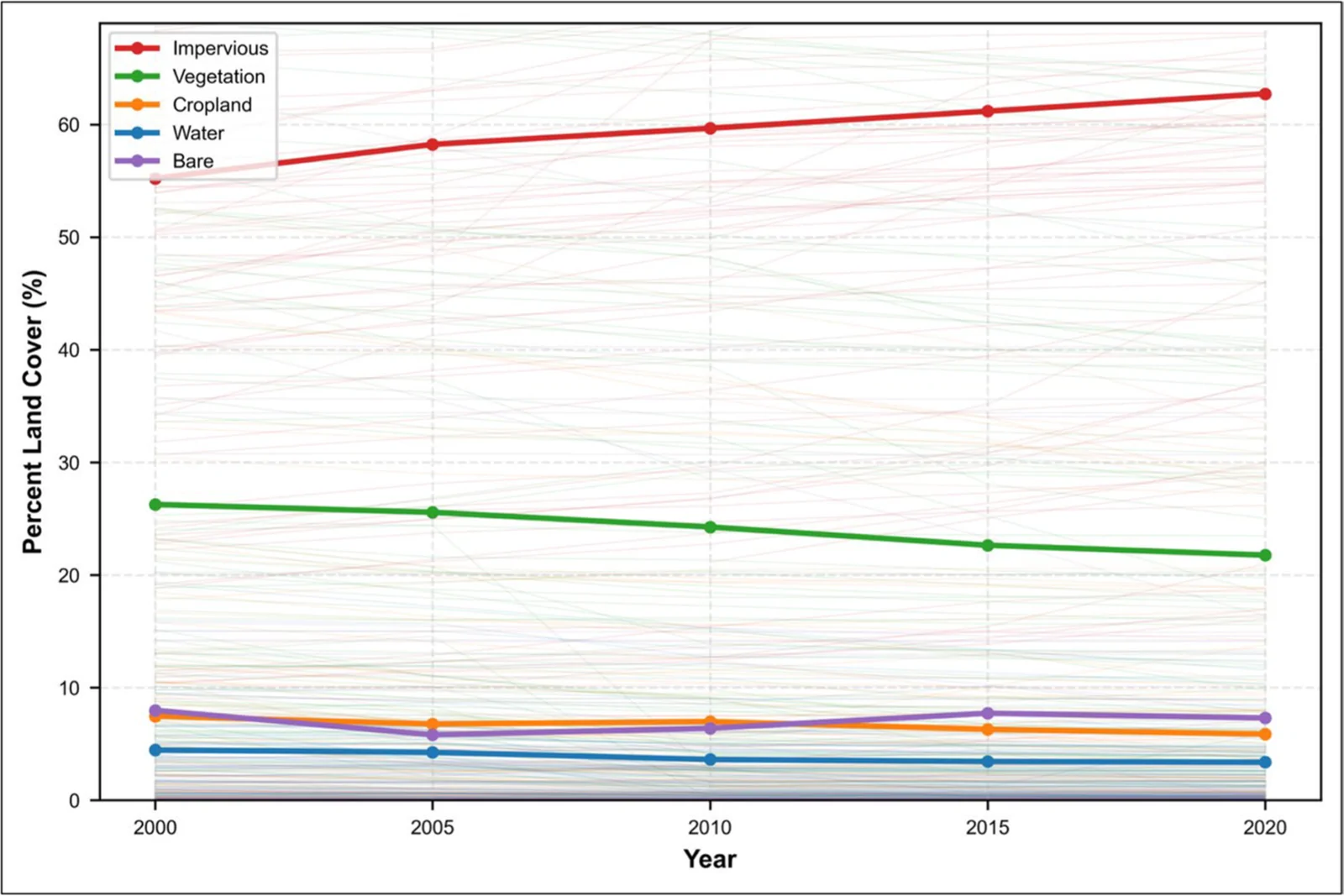
LC composition trends across 100 global cities (2000–2020). Bold lines indicate the mean percent cover for each LC class averaged across all cities; thin lines show individual city trajectories. Color coding follows Figure 3

**Fig. 5:**
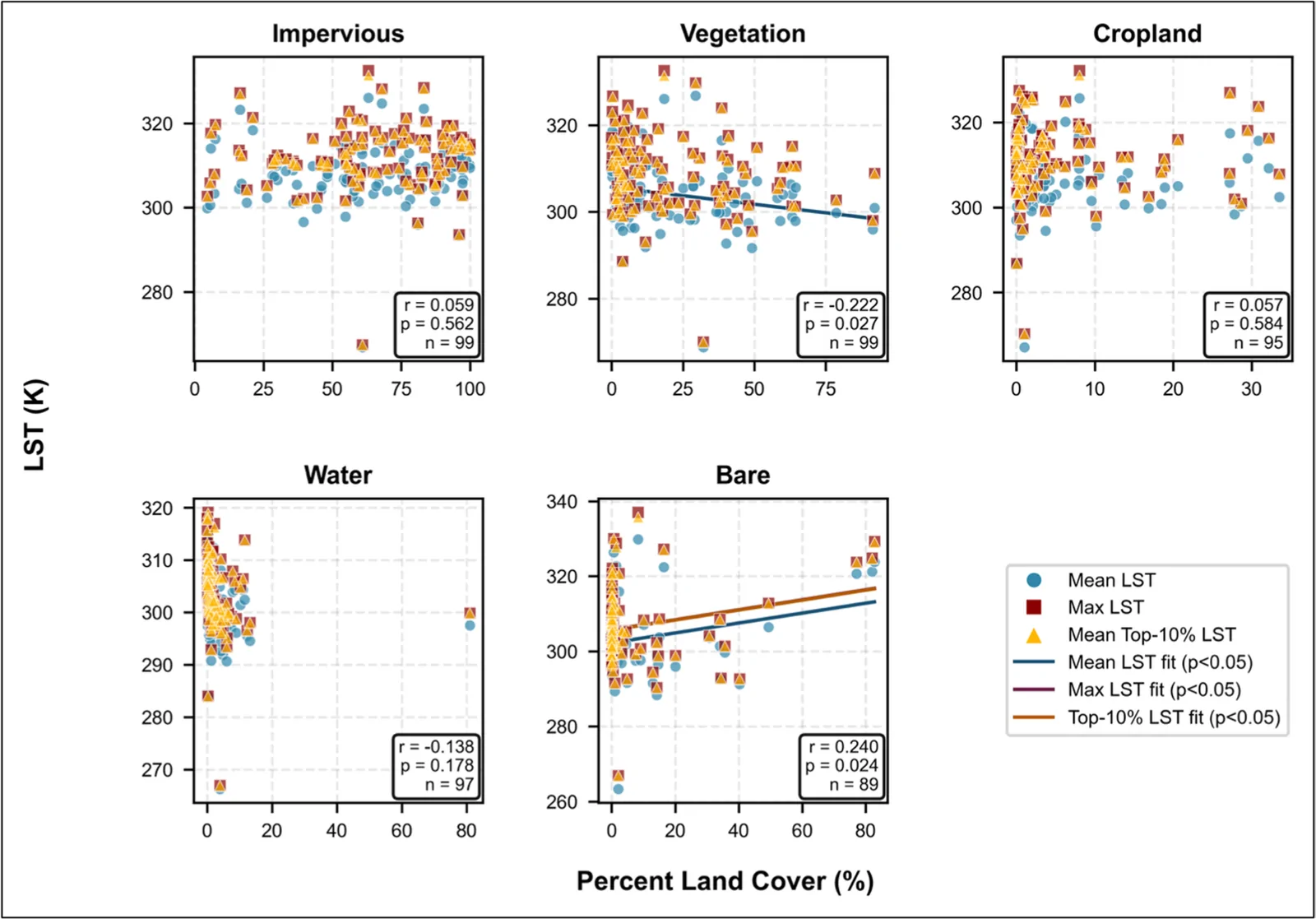
Relationships between percent cover of five major LC classes and mean summer LST in 100 cities (2020). Each point represents one city. Blue circles = mean LST; red squares = $LST_{\text{Max}}$; yellow triangles = $LST_{\text{Max}10}$. Solid lines indicate significant linear fits (p < 0.05)

**Fig. 6:**
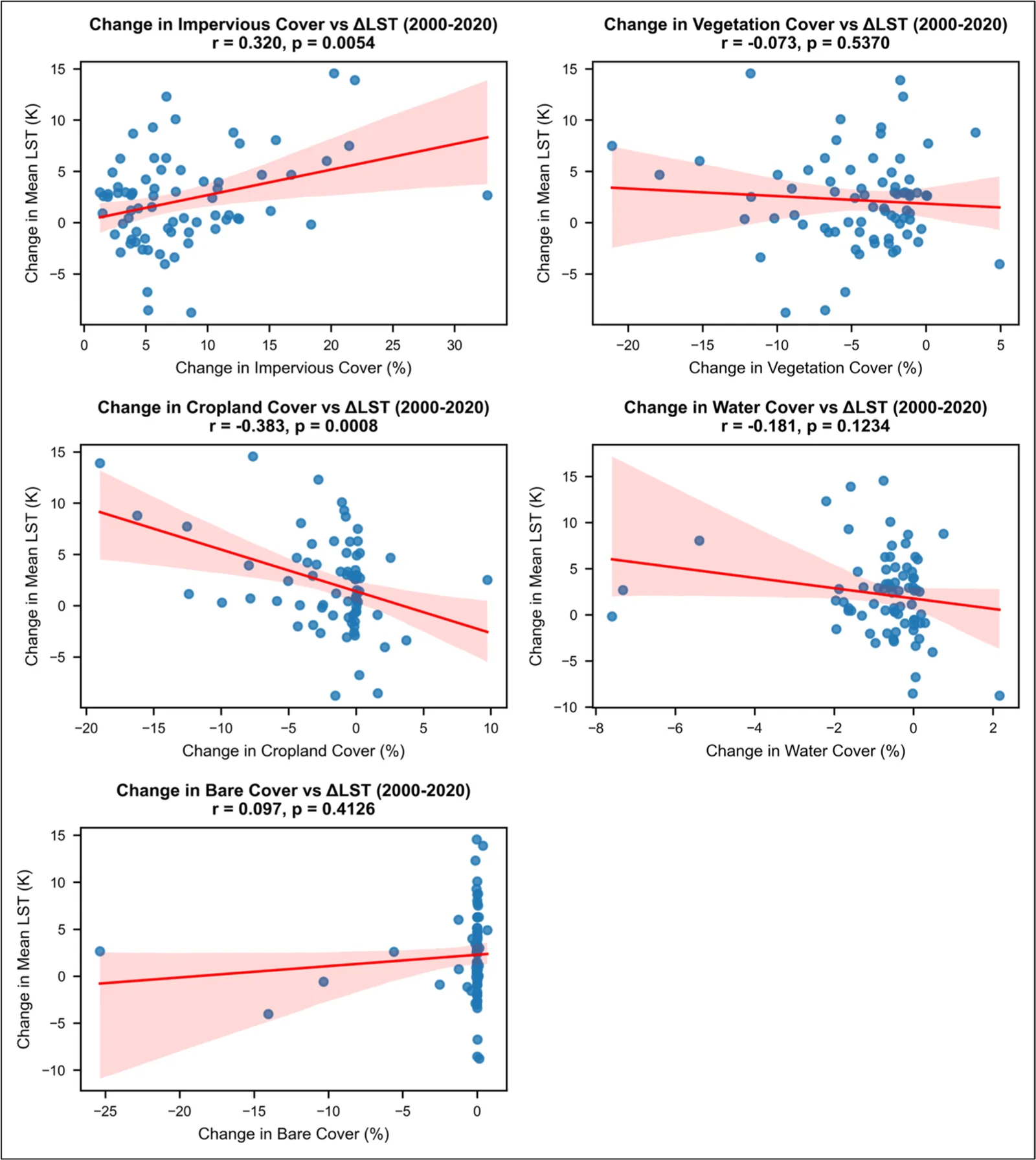
Bidirectional analysis of LC changes and LST dynamics. Each point represents one city. Red lines indicate significant linear fits (p < 0.05). Only ΔLST is shown; results for extreme heat metrics ($\Delta LST_{\text{Max}}$, and $\Delta LST_{\text{Max}10}$) are reported in Table 4

**Fig. 7:**
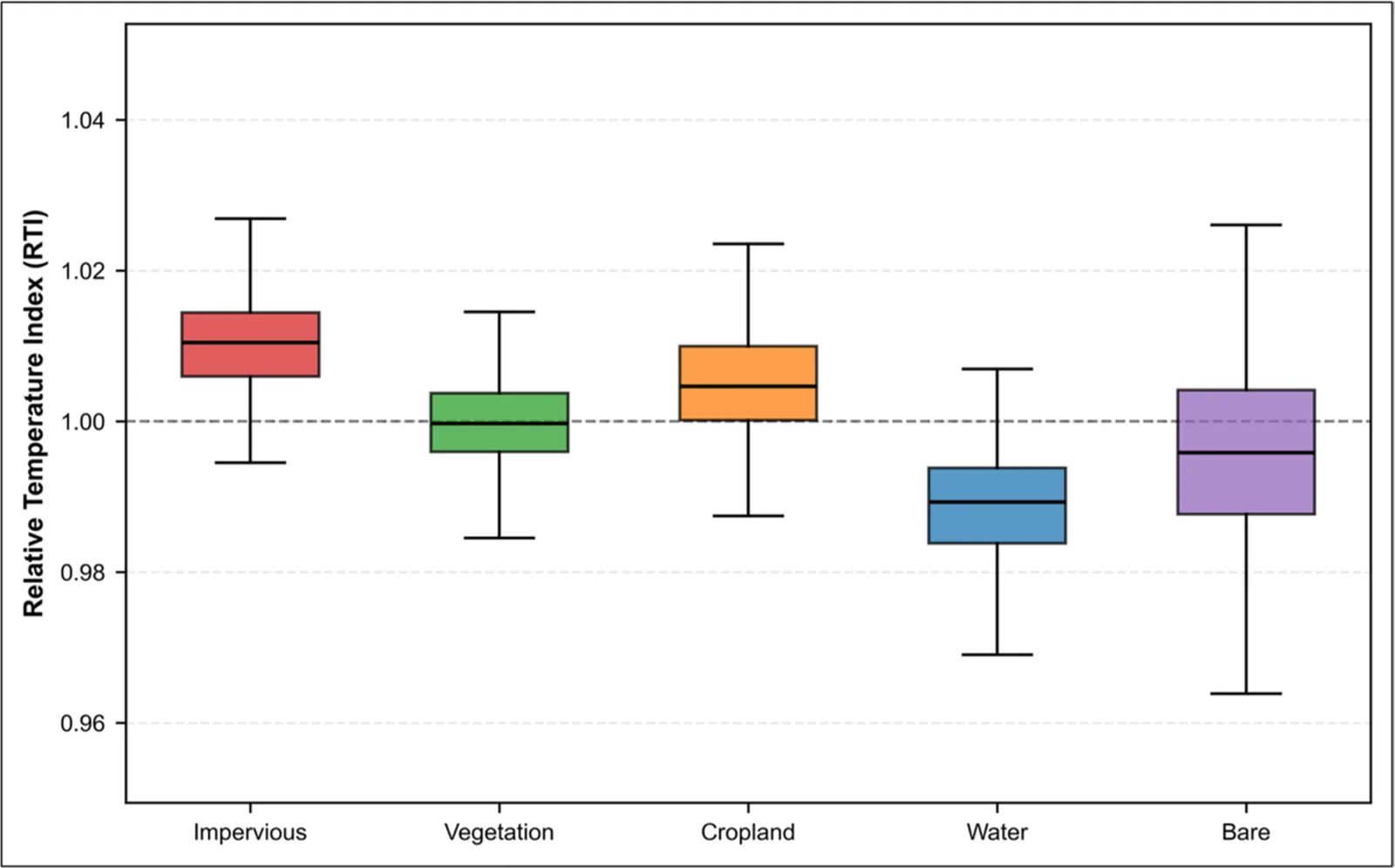
Distribution of RTI values by LC class across all city-years (n = 2,244). Boxes show the interquartile range with the median line; whiskers extend to 1.5× the interquartile range. The dashed line at RTI = 1.0 indicates thermal parity with the city-year mean

**Fig. 8:**
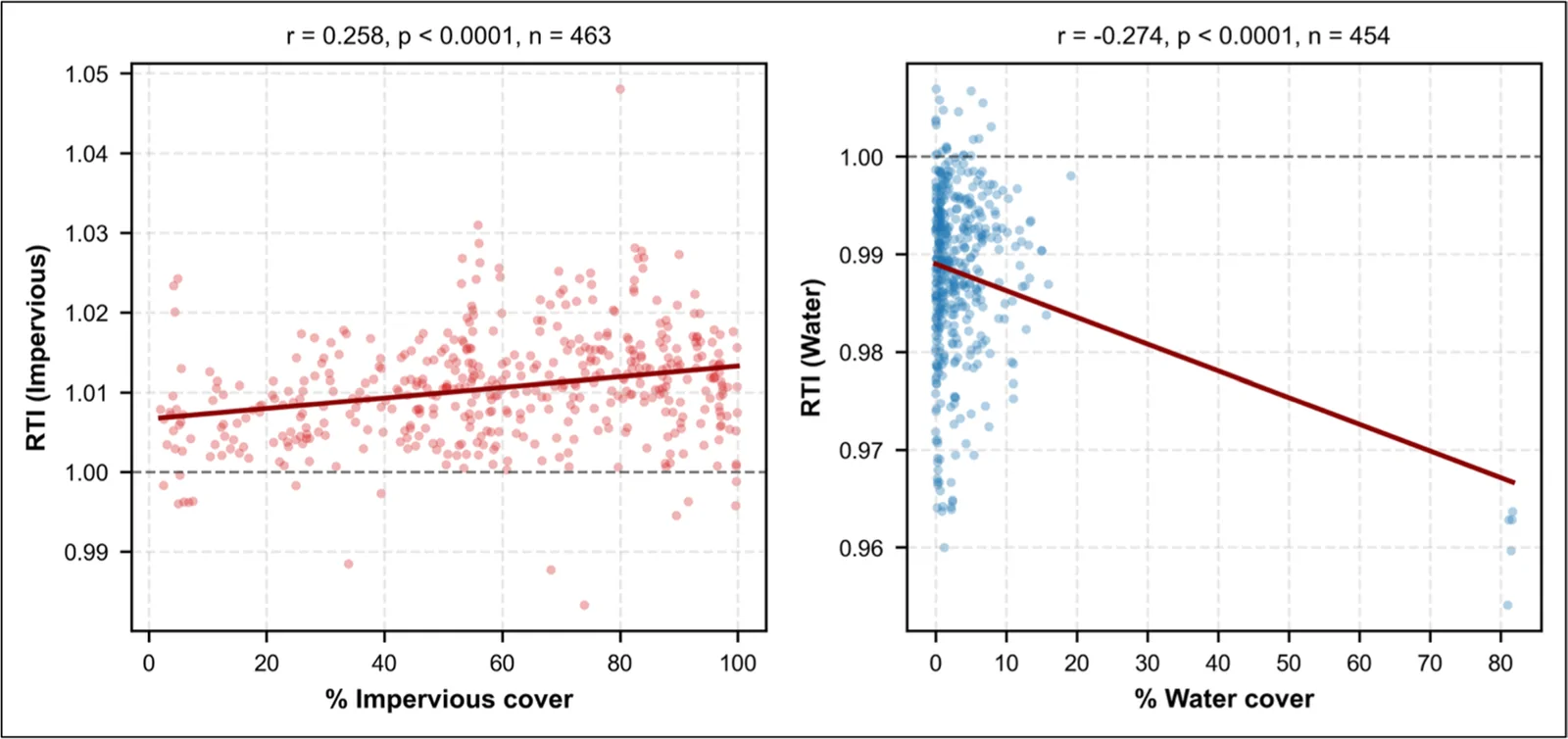
Sensitivity of RTI to LC composition. (**a**) RTI for impervious surfaces versus percent impervious cover per city-year (r = 0.258, p < 0.0001, n = 463). (**b**) RTI for water versus percent water cover per city-year (r = −0.274, p < 0.0001, n = 454). Red lines show linear fits; dashed lines indicate RTI = 1.0

**Table 1:** Reclassification of GLAD LC Classes for UHI analysis

LC Class	Abbreviation	GLAD Class Code(s)	Description
Impervious	imp	250	Built-up areas including concrete, asphalt, roof-tops, and other artificial surfaces associated with urban infrastructure.
Vegetation	veg	4–48, 104–124, 125–148	Natural and semi-natural vegetation including forests, shrublands, wetlands, and wetland tree cover.
Cropland	crop	244	Agricultural lands including annual crops, orchards, plantations, and other cultivated areas.
Water	wat	200–207, 254, 241	Open water bodies, snow/ice cover, and seasonal water features.
Bare	bare	0–3, 100–103	Areas with sparse or no vegetation, including exposed soil, rock, sand, and barren terrain.

**Table 2:** Summary metrics for city-level LST analysis

Metric	Variable	Aspect of population heat exposure represented
Mean LST	LST	Average surface temperature exposure
Max LST	LST_Max_	Hottest locations; potential heat risk hotspots
Upper-Decile Mean LST	LST_Max10_	Frequency/intensity of extreme heat exposure

**Table 3:** Pearson correlation coefficients (r), R^2^, and p-values for the relationship between percent cover of each LC class and LST, $LST_{\text{Max}},LST_{\text{Max}10}$ in 2020 across 100 cities

LC	LST r	LST P	LST p(BH)	${\text{LST}}_{\text{Max}}$ r	${\text{LST}}_{\text{Max}}$ p	${\text{LST}}_{\text{Max}}$ p(BH)	${\text{LST}}_{\text{Max10}}$ r	${\text{LST}}_{\text{Max10}}$ p	${\text{LST}}_{\text{Max10}}$ p(BH)
Imp	+0.0589	+0.5624	+0.5875	+0.0561	+0.5814	+0.5875	+0.0552	+0.5875	+0.5875
Veg	−0.2220	+**0.0272**	+0.1350	−0.1798	+0.0749	+0.1906	−0.1790	+0.0762	+0.1906
Crop	+0.0569	+0.5837	+0.5875	+0.0621	+0.5497	+0.5875	+0.0639	+0.5382	+0.5875
Wat	−0.1378	+0.1784	0.2974	−0.1462	+0.1529	+0.2898	−0.1457	+0.1546	+0.2898
Bare	+0.2397	+**0.0237**	0.1350	+0.2226	+**0.0360**	+0.1350	+0.2236	+**0.0352**	+0.1350

Benjamini-Hochberg-corrected p-values (15 tests, α=0.05) are reported alongside uncorrected values, no correlation remains significant after correction

**Table 4:** Pearson correlation coefficients $(r),R^{2}$, and p-*values* for the relationship between net LC change (2000–2020) and change in LST, ${\text{LST}}_{\text{Max}}$, and $LST_{\text{Max}10}$ across cities

LC	ΔLST r	ΔLST p	ΔLST P(BH)	$\Delta {\text{LST}}_{\text{Max}}$ r	$\Delta {\text{LST}}_{\text{Max}}$ p	$\Delta {\text{LST}}_{\text{Max}}$ P(BH)	$\Delta {\text{LST}}_{\text{Max10}}$ r	$\Delta {\text{LST}}_{\text{Max10}}$ p	$\Delta {\text{LST}}_{\text{Max10}}$ P(BH)
imp	+0.3203	+**0.0054**	+**0.0372**	+0.2980	+**0.0099**	+**0.0372**	+0.2996	+**0.0095**	+**0.0372**
veg	−0.0729	+0.5370	+0.5370	−0.1221	+0.3000	+0.4398	−0.1166	+0.3225	+0.4398
crop	−0.3826	+**0.0008**	+**0.0115**	−0.2655	+**0.0222**	+0.0555	−0.2683	+**0.0208**	+0.0555
wat	−0.1807	+0.1234	+0.2057	−0.2448	+**0.0355**	+0.0666	−0.2510	+**0.0310**	+0.0664
bare	+0.0967	+0.4126	+0.5158	+0.0816	+0.4897	+0.5370	+0.0777	+0.5103	+0.5370

Benjamini-Hochberg-corrected (BH) p-values (15 tests, α=0.05) are reported alongside uncorrected values. A stricter Bonferroni correction preserves only the cropland ΔLST result, see Table 5 for the primary multivariate evidence underlying the impervious and cropland findings

**Table 5:** Multiple linear regression results predicting city-level change in ΔLST, $\Delta {\text{LST}}_{\text{Max}}$, and $\Delta {\text{LST}}_{\text{Max}10}$ between 2000 and 2020 from net percentage changes in the area of each LC class, plus two boundary-sensitivity covariates (area ratio, perimeter-area ratio). Positive coefficients indicate warming associated with expansion of the corresponding LC class or increase in the covariate

LC Class	ΔLST β	ΔLST p	$\Delta {\text{LST}}_{\text{Max}}$ β	$\Delta {\text{LST}}_{\text{Max}}$ p	$\Delta {\text{LST}}_{\text{Max}10}$ β	$\Delta {\text{LST}}_{\text{Max}10}$ p
$\Delta \%LC_{\text{imp},00\to 20}$	+0.1884	+**0.0096**	+0.2989	+**0.0021**	+0.3005	+**0.0019**
$\Delta \%LC_{\text{veg},00\to 20}$	−0.0446	+0.6634	−0.0881	+0.5164	−0.0769	+0.5688
$\Delta \%LC_{\text{crop},00\to 20}$	−0.2072	+**0.0398**	−0.1022	+0.4368	−0.1022	+0.4342
$\Delta \%LC_{\text{wat},00\to 20}$	−0.0881	+0.7498	−0.3824	+0.2975	−0.4037	+0.2689
$\Delta \%LC_{\text{bare},00\to 20}$	+0.1515	+0.2800	+0.2737	+0.1417	+0.2822	+0.1279
area_ratio	−0.0134	+0.8656	−0.0545	+0.6036	−0.0476	+0.6476
Perimeter_area_ratio	−0.1592	+0.1945	−0.2350	+0.1485	−0.2300	+0.1549

**Table 6:** Pixel-level direct thermal responses by LC transition type (2000–2020), grouped by source LC class (2000) in the order impervious, vegetation, cropland, water, bare; destination classes (2020) follow the same order within each group. n_cities indicates the number of cities in which each transition was observed; transitions with n≤5 (bolded) are rare and should be interpreted with caution

Transition	n_cities	Mean ΔLST	$\Delta {\text{LST}}_{\text{Max}}$	${\text{LST}}_{\text{Max10}}$
imp→veg	**5**	+1.2297	−0.3108	−0.3108
imp→crop	**2**	−0.0762	−0.7524	−0.7524
imp→wat	**3**	+1.1467	+0.8625	+0.8625
imp→bare	**3**	+3.5219	+0.5598	+0.5442
veg→imp	91	+2.8571	+4.8059	+4.7568
veg→crop	85	+2.3014	+4.8643	+4.8042
veg→wat	86	+0.5732	+1.9174	+1.8771
veg→bare	67	+1.2964	+2.7398	+2.6872
crop→imp	84	+3.2462	+5.1415	+5.0921
crop→veg	81	+2.4205	+4.5023	+4.4470
crop→wat	60	−0.4102	+0.5982	+0.5522
crop→bare	30	+2.7213	+2.7968	+2.7126
wat→imp	87	+4.1619	+6.0686	+6.0251
wat→veg	84	+3.7427	+5.8891	+5.8514
wat→crop	54	+3.8838	+6.3919	+6.4235
wat→bare	72	+4.9336	+6.6669	+6.6301
bare→imp	77	+3.0448	+4.4748	+4.4518
bare→veg	54	+2.2571	+3.9933	+3.9186
bare→crop	25	+2.2873	+3.2878	+3.1846
bare→wat	68	+0.2139	+0.7493	+0.7068

**Table 7:** RTI statistics by LC type across all city-years (n=2244). Min and max values represent 10th and 90th percentiles, respectively, to reduce mixed-pixel effects

LC Class	Mean	SD	Min	Max	Mode
imp	1.0106	0.0069	1.0028	1.0189	0.9833
veg	1.0000	0.0065	0.9923	1.0082	0.9773
crop	1.0054	0.0080	0.9966	1.0164	0.9841
wat	0.9880	0.0088	0.9768	0.9976	0.9541
bare	0.9956	0.0130	0.9785	1.0130	0.9621

**Table 8:** Evidence-based urban planning recommendations derived from LC-ΔLST regression and RTI analyses

Recommendation	Supporting evidence
Limit impervious expansion	Impervious expansion is the strongest city-scale predictor of warming (β=+0.1884) and the strongest predictor of extreme-heat increases (+4.5–6.0 K for $\Delta LST_{\text{Max}10}$).
Prioritize imp→veg/wat conversions	De-sealing transitions were too rare (n≤5 cities) to quantify directly, but the consistent warming from all impervious conversions supports de-sealing as a theoretically sound strategy.
Preserve vegetation and water	Water bodies show the strongest cooling signature (RTI=0.9880); water loss is associated with the largest LST increases of any transition type (+3.7–4.9 K mean; up to +6.7 K for $\Delta LST_{\text{Max}}$).
Evaluate mitigation using extreme-heat metrics, not mean temperature	Extreme-heat responses to impervious expansion (1.5–1.7×) and water loss (≈1.6×) are systematically larger than mean ΔLST responses, so mean-temperature-only evaluation understates real benefits.

## Data Availability

All data and code necessary to reproduce the results are available in a public GitHub repository: https://github.com/SaghiSarafi/urban_heat_landcover. The repository includes the full GEE scripts, Python analysis scripts, and derived datasets (city-year LST summaries and pixel-level transition tables). Land cover data were obtained from the GLAD global LC dataset (Potapov et al. 2022), Landsat imagery was accessed via GEE (Gorelick et al. 2017), and global mean land-only surface temperature was obtained from Berkeley Earth (Rohde and Hausfather 2020).

## References

[R1] Almeida CRD, Teodoro AC, Gonçalves A (2021) Study of the Urban Heat Island (UHI) Using Remote Sensing Data/Techniques: A Systematic Review. Environments 8:105. 10.3390/environments8100105

[R2] Benjamini Y, Hochberg Y (1995) Controlling the False Discovery Rate: A Practical and Powerful Approach to Multiple Testing. J Royal Stat Soc Ser B: Stat Methodol 57:289–300. 10.1111/j.2517-6161.1995.tb02031.x

[R3] Boeing G (2017) OSMnx: New methods for acquiring, constructing, analyzing, and visualizing complex street networks. Comput Environ Urban Syst 65:126–139. 10.1016/j.compenvurbsys.2017.05.004

[R4] Chakraborty T, Hsu A, Manya D, Sheriff G (2019) Disproportionately higher exposure to urban heat in lower-income neighborhoods: a multi-city perspective. Environ Res Lett 14:105003. 10.1088/1748-9326/ab3b99

[R5] Chen X-L, Zhao H-M, Li P-X, Yin Z-Y (2006) Remote sensing image-based analysis of the relationship between urban heat island and land use/cover changes. Remote Sens Environ 104:133–146. 10.1016/j.rse.2005.11.016

[R6] Chen H, Deng Q, Zhou Z (2022) Influence of land cover change on spatio-temporal distribution of urban heat island —a case in Wuhan main urban area. Sustain Cities Soc 79:103715. 10.1016/j.scs.2022.103715

[R7] Chen C, Li Y, Wang X (2024) Biophysical effects of croplands on land surface temperature. Nat Commun 15:10901. 10.1038/s41467-024-55319-2 PMC11685707 39738094

[R8] Climate Adaptation Platform (2024) Urban heat island effects amplify climate change impact. https://climateadaptationplatform.com/urban-heat-island-effects-amplify-climate-change-impact/.Accessed 9 Feb 2026

[R9] Creutzig F, Baiocchi G, Bierkandt R (2015) Global typology of urban energy use and potentials for an urbanization mitigation wedge. Proc Natl Acad Sci USA 112:6283–6288. 10.1073/pnas.1315545112 PMC4443311 25583508

[R10] U.S. Environmental Protection Agency (2025) Reducing urban heat islands: Compendium of strategies. https://www.epa.gov/heat-islands/heat-island-compendium. Accessed 11 July 2026

[R11] Ermida SL, Soares P, Mantas V (2020) Google Earth Engine Open-Source Code for Land Surface Temperature Estimation from the Landsat Series. Remote Sens 12:1471. 10.3390/rs12091471

[R12] Fick SE, Hijmans RJ (2017) WorldClim 2: new 1-km spatial resolution climate surfaces for global land areas. Intl J Climatology 37:4302–4315. 10.1002/joc.5086

[R13] Geletič J, Lehnert M, Savić S, Milošević D (2019) Inter-/intra-zonal seasonal variability of the surface urban heat island based on local climate zones in three central European cities. Build Environ 156:21–32. 10.1016/j.buildenv.2019.04.011

[R14] Gorelick N, Hancher M, Dixon M (2017) Google Earth Engine: Planetary-scale geospatial analysis for everyone. Remote Sens Environ 202:18–27. 10.1016/j.rse.2017.06.031

[R15] Guo Y, Gasparrini A, Armstrong BG (2017) Heat Wave and Mortality: A Multicountry, Multicommunity Study. Environ Health Perspect 125:087006. 10.1289/EHP1026 PMC5783630 28886602

[R16] Heo S, Bell ML (2023) Investigation on urban greenspace in relation to sociodemographic factors and health inequity based on different greenspace metrics in 3 US urban communities. J Expo Sci Environ Epidemiol 33:218–228. 10.1038/s41370-022-00468-z PMC10005950 35995844

[R17] Hoffman JS, Shandas V, Pendleton N (2020) The Effects of Historical Housing Policies on Resident Exposure to Intra-Urban Heat: A Study of 108 US Urban Areas. Climate 8:12. 10.3390/cli8010012

[R18] Islam HE-G, Noha SD, Hala AE (2021) Monitoring the urban heat island phenomenon using thermal infrared bands of Landsat images from 1998 to 2018, El-Senbellaween City, Egypt. J Environ Sci 50:287–323. 10.21608/jes.2021.223394

[R19] Kalnay E, Cai M (2003) Impact of urbanization and land-use change on climate. Nature 423:528–531. 10.1038/nature01675 12774119

[R20] Khyami A (2022) Impact of land cover change on land surface temperature over Greater Beirut Area – Lebanon. JASTT 3:74–80. 10.38094/jastt302174

[R21] Lobell DB, Bonfils C, Faurès J-M (2008) The Role of Irrigation Expansion in Past and Future Temperature Trends. Earth Interact 12:1–11. 10.1175/2007EI241.1

[R22] Lüthi S, Fairless C, Fischer EM (2023) Rapid increase in the risk of heat-related mortality. Nat Commun 14:4894. 10.1038/s41467-023-40599-x PMC10449849 37620329

[R23] Mildrexler DJ, Zhao M, Running SW (2011) A global comparison between station air temperatures and MODIS land surface temperatures reveals the cooling role of forests. J Geophys Res 116:G03025. 10.1029/2010JG001486

[R24] Morice CP, Kennedy JJ, Rayner NA (2021) An Updated Assessment of Near-Surface Temperature Change From 1850: The HadCRUT5 Data Set. JGR Atmos 126. 10.1029/2019JD032361. e2019JD032361

[R25] NASA (2022) MODIS land surface temperature products (MOD11). In: NASA Earth Science Data Systems. https://modis.gsfc.nasa.gov/data/dataprod/mod11.php

[R26] National Oceanic and Atmospheric Administration (2024) Understanding ENSO: El Niño and La Niña. In: Climate.gov. https://www.climate.gov. Accessed 9 Feb 2026

[R27] OpenStreetMap contributors (2024) OpenStreetMap Data. https://www.openstreetmap.org/

[R28] Peng S, Piao S, Ciais P (2012) Surface Urban Heat Island Across 419 Global Big Cities. Environ Sci Technol 46:696–703. 10.1021/es2030438 22142232

[R29] Potapov P, Hansen MC, Pickens A (2022) The Global 2000–2020 Land Cover and Land Use Change Dataset Derived From the Landsat Archive: First Results. Front Remote Sens 3:856903. 10.3389/frsen.2022.856903

[R30] Ren J, Shi K, Li Z (2023) A Review on the Impacts of Urban Heat Islands on Outdoor Thermal Comfort. Buildings 13:1368. 10.3390/buildings13061368

[R31] Revi A, Satterthwaite D, Aragón-Durand F (2014) Towards transformative adaptation in cities: the IPCC’s Fifth Assessment. Environ Urban 26:11–28. 10.1177/0956247814523539

[R32] Ritchie H, Roser M, Ortiz-Ospina E (2025) Population with UN projections. https://ourworldindata.org/grapher/population-with-un-projections

[R33] Rivera A, Moore N, Kim JH (2024) Evapotranspiration Impacts on Summer Surface Urban Heat Island Distributions and Trends in Santa Clara, California: Moderating Effects of the Human Environment. In: Roggema R (ed) Dry Urbanism. Springer Nature Switzerland, Cham, pp 235–259

[R34] Rohde RA, Hausfather Z (2020) The Berkeley Earth Land/Ocean Temperature Record. Earth Syst Sci Data 12:3469–3479. 10.5194/essd-12-3469-2020

[R35] Schiavina M, Melchiorri M, Pesaresi M (2023) GHS-SMOD R2023A-GHS settlement layers, application of the Degree of Urbanisation methodology (stage I) to GHS-POP R2023A and GHS-BUILT-S R2023A, multitemporal (1975–2030). 10.2905/A0DF7A6F-49DE-46EA-9BDE-563437A6E2BA

[R36] Seabold S, Perktold J (2010) Statsmodels: Econometric and statistical modeling with Python. In: Proceedings of the 9th Python in Science Conference. p 61

[R37] Seneviratne SI, Corti T, Davin EL (2010) Investigating soil moisture-climate interactions in a changing climate: A review. Earth Sci Rev 99:125–161. 10.1016/j.earscirev.2010.02.004

[R38] Shahmohamadi P, Che-Ani AI, Maulud KNA (2011) The Impact of Anthropogenic Heat on Formation of Urban Heat Island and Energy Consumption Balance. Urban Stud Res 2011:497524. 10.1155/2011/497524

[R39] Small C (2003) High spatial resolution spectral mixture analysis of urban reflectance. Remote Sens Environ 88:170–186. 10.1016/j.rse.2003.04.008

[R40] United Nations, Department of Economic and Social Affairs, Division P (2018) World urbanization prospects: The 2018 revision. https://esa.un.org/unpd/wup/. Accessed 11 July 2026

[R41] Venter ZS, Chakraborty T, Lee X (2021) Crowdsourced air temperatures contrast satellite measures of the urban heat island and its mechanisms. Sci Adv 7:eabb9569. 10.1126/sciadv.abb9569 PMC8153720 34039596

[R42] Wang X, Li H, Sodoudi S (2022) The effectiveness of cool and green roofs in mitigating urban heat island and improving human thermal comfort. Build Environ 217:109082. 10.1016/j.buildenv.2022.109082

[R43] Weng Q (2012) Remote sensing of impervious surfaces in the urban areas: Requirements, methods, and trends. Remote Sens Environ 117:34–49. 10.1016/j.rse.2011.02.030

[R44] Wu C, Murray AT (2003) Estimating impervious surface distribution by spectral mixture analysis. Remote Sens Environ 84:493–505. 10.1016/S0034-4257(02)00136-0

[R45] Zhang P, Ren G, Qin Y (2021) Urbanization Effects on Estimates of Global Trends in Mean and Extreme Air Temperature. J Clim 34:1923–1945. 10.1175/JCLI-D-20-0389.1

[R46] Zhang H, Yin Y, An H (2022) Surface urban heat island and its relationship with land cover change in five urban agglomerations in China based on GEE. Environ Sci Pollut Res 29:82271–82285. 10.1007/s11356-022-21452-y 35750907

[R47] Zhang X, Zhao T, Xu H (2024) GLC_FCS30D: the first global 30 m land-cover dynamics monitoring product with a fine classification system for the period from 1985 to 2022 generated using dense-time-series Landsat imagery and the continuous change-detection method. Earth Syst Sci Data 16:1353–1381. 10.5194/essd-16-1353-2024

[R48] Zhao L, Lee X, Smith RB, Oleson K (2014) Strong contributions of local background climate to urban heat islands. Nature 511:216–219. 10.1038/nature13462 25008529

[R49] Zhou D, Zhao S, Liu S (2014) Surface urban heat island in China’s 32 major cities: Spatial patterns and drivers. Remote Sens Environ 152:51–61. 10.1016/j.rse.2014.05.017

[R50] Zipper SC, Schatz J, Kucharik CJ, Loheide SP (2017) Urban heat island-induced increases in evapotranspirative demand. Geophys Res Lett 44:873–881. 10.1002/2016GL072190

[R51] Zomer RJ, Trabucco A, Bossio DA, Verchot LV (2008) Climate change mitigation: A spatial analysis of global land suitability for clean development mechanism afforestation and reforestation. Agric Ecosyst Environ 126:67–80. 10.1016/j.agee.2008.01.014

